# ARRB1 suppresses the activation of hepatic macrophages via modulating endoplasmic reticulum stress in lipopolysaccharide-induced acute liver injury

**DOI:** 10.1038/s41420-021-00615-9

**Published:** 2021-08-28

**Authors:** Yiming Lei, Sizhe Wan, Huiling Liu, Haoxiong Zhou, Lingjun Chen, Yidong Yang, Bin Wu

**Affiliations:** 1 Department of Gastroenterology, The Third Affiliated Hospital of Sun Yat-Sen University, Guangzhou, Guangdong Province China; 2 Guangdong Provincial Key Laboratory of Liver Disease Research, Guangzhou, Guangdong Province China

**Keywords:** Molecular biology, Chemical biology, Physiology

## Abstract

Acute liver injury (ALI) caused by multiple inflammatory responses is a monocyte-/macrophage-mediated liver injury that is associated with high morbidity and mortality. Liver macrophage activation is a vital event that triggers ALI. However, the mechanism of liver macrophage activation has not been fully elucidated. This study examined the role of β-arrestin1 (ARRB1) in wild-type (WT) and *ARRB1-*knockout (*ARRB1*-KO) mouse models of ALI induced by lipopolysaccharide (LPS), and *ARRB1*-KO mice exhibited more severe inflammatory injury and liver macrophage activation compared to WT mice. We found that LPS treatment reduced the expression level of ARRB1 in Raw264.7 and THP-1 cell lines, and mouse primary hepatic macrophages. Overexpression of *ARRB1* in Raw264.7 and THP-1 cell lines significantly attenuated LPS-induced liver macrophage activation, such as transformation in cell morphology and enhanced expression of proinflammatory cytokines (tumor necrosis factor-α, interleukin-1β, and interleukin-6), while downregulation of *ARRB1* by small interfering RNA and *ARRB1* deficiency in primary hepatic macrophages both aggravated macrophage activation. Moreover, overexpression of *ARRB1* suppressed LPS-induced endoplasmic reticulum (ER) stress in liver macrophages, and inhibition of ER stress impeded excessive hepatic macrophage activation induced by downregulation of ARRB1. Our data demonstrate that ARRB1 relieves LPS-induced ALI through the ER stress pathway to regulate hepatic macrophage activation and that ARRB1 may be a potential therapeutic target for ALI.

## Introduction

Acute liver injury (ALI) is a complex and life-threatening disease caused by multiple inflammation-related etiologies, such as sepsis, alcohol addiction, metabolic syndrome, drug-induced liver disease, and virus or bacterial infection [1, 2], and is associated with high mortality due to a lack of specific therapy. Despite considerable differences in these etiologies, the pathophysiological manifestations of ALI are mainly hepatocyte injury, inflammatory cell infiltration and activation, and disordered inflammatory response [3]. In this pathologic process, liver macrophages (Kupffer cells) play a crucial role as modulators of the immune system [4], and activated macrophages regulate the immune response by recognizing and expressing antigens and releasing inflammatory cytokines [5]. Activated liver macrophages display increased size, morphology of polygons, pseudopodia, and enhanced expression of inflammatory cytokines, leading to amplification of the inflammatory response cascade and sustaining hepatocyte damage [6]. Hence, controlling overactivated liver macrophages is a potentially crucial strategy for preventing and curing ALI. Previous studies have confirmed that the performance of lipopolysaccharide (LPS), a well-known inflammatory ligand, can establish experimental mouse ALI models [7]. LPS from gram-negative bacteria can activate macrophages to release a variety of inflammatory cytokines including tumor necrosis factor-α (TNF-α), interleukin-1β (IL-1β), and IL-6, which in turn lead to massive hepatocyte apoptosis or necrosis along with aspartate aminotransferase (AST) and alanine aminotransferase (ALT) release into peripheral blood [8].

β-Arrestin1 (ARRB1) belongs to the arrestin family originally identified as a multifunctional adaptor protein that negatively regulates the desensitization and internalization of G-protein-coupled receptors (GPCRs) [9]. Recent studies have also indicated that ARRB1, as a mediator rather than simply a terminator of GPCR signaling, can regulate G-protein-independent signaling cascades or act as a scaffold to connect various receptors to downstream signaling pathways, such as the c-Jun N-terminal kinase and nuclear factor-κB (NF-κB) cascades [10, 11]. In addition, ARRB1 is closely related to the inflammatory response. It was reported that in LPS/ATP-stimulated BV2 microglia, overexpression of ARRB1 attenuated the inhibitory effect of a specific nicotinic acetylcholine receptor agonist on inflammasome activation [12]. Deficiency of ARRB1 abolished prostaglandin E2-elicited suppression of IL-10 production in LPS-elicited inflammatory conditions [13]. Sharma et al. revealed that ARRB1 in nonhematopoietic cells was critical and sufficient for inhibiting sepsis-induced inflammation [14]. Nevertheless, there are few reports about the regulation of ARRB1 on macrophage activation in the inflammatory microenvironment. ARRB1 plays a crucial role in the occurrence and development of numerous diseases. In our previous study, we found that ARRB1 is involved in hepatocellular carcinogenesis through inflammation-mediated Akt signaling [15] and the pathological progression of portal hypertensive gastropathy [16]. However, our knowledge of the function of ARRB1 in ALI is limited.

Endoplasmic reticulum (ER) stress is a pathological status of subcellular organelles triggered by misfolded and unfolded protein aggregation and Ca^2+^ balance disorder in the ER lumen, and has long been considered to have a promoting role in ALI. Emerging evidence has indicated that ER stress signaling has close cross-talk with macrophage activation [17]. Wei et al. reported that secreted Golgi protein 73 from tumor cells stimulated activation of neighboring macrophages in the liver to release cytokines and chemokines via ER stress [18]. In addition, ARRB1 was reported to protect against ER stress/PUMA-induced mucosal epithelial apoptosis in portal hypertensive gastropathy [19].

In our present study, we demonstrated that *ARRB1* deficiency aggravated LPS-induced ALI, and overexpression of *ARRB1* suppressed liver macrophage activation and the release of inflammatory cytokines. Inhibition of ER stress attenuated more severe ALI and liver macrophage activation induced by downregulation of ARRB1. Overall, our findings suggest that ARRB1 protects against LPS-induced ALI via ER stress and that targeting ARRB1-mediated ER stress may provide a potential method for ALI therapy.

## Results

### LPS induces ALI along with activation of hepatic macrophages

**Fig. 1:**
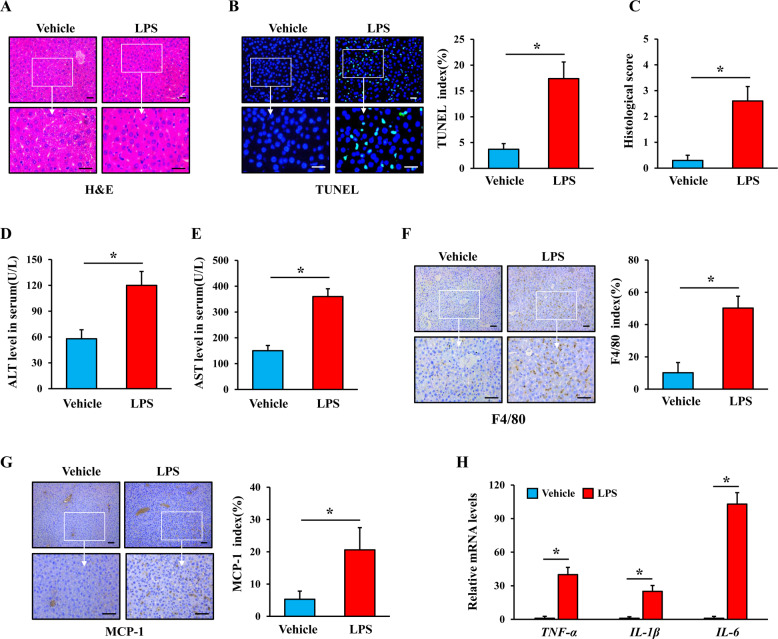
LPS induced acute liver injury and hepatic macrophage infiltration. Mice were intraperitoneally administered either physiological saline (vehicle) or a single LPS injection (5 mg/kg), and the mouse livers were harvested at 6 h. **A** Representative H&E staining of livers in vehicle- and LPS-induced WT mice (scale bar: 25 μm). **B** TUNEL staining of livers in vehicle- and LPS-induced mice (scale bar: 25 μm), and the positive TUNEL index was scored. **C** Liver histology scoring in vehicle- and LPS-induced mice. **D**, **E** Serum ALT and AST levels in vehicle- and LPS-induced mice. **F**, **G** F4/80 and MCP-1 staining of livers in vehicle- and LPS-induced mice (scale bar: 50 μm), and the positive F4/80 and MCP-1 indexes were scored. **H** *TNF-α*, *IL-1β*, and *IL-6* mRNA levels in vehicle- and LPS-induced WT mouse liver tissues were analyzed by real-time PCR. All values are the mean ± SD (*n* = 6 in each group). *P < 0.05 by Student’s *t* test.

LPS is the part of the outer membranes of gram-negative bacteria, which induces multiple inflammatory responses. In this study, wild-type (WT) mice were intraperitoneally administered either physiological saline or a single LPS injection (5 mg/kg) to induce ALI, and the mouse livers were harvested after 6 h. Hematoxylin and eosin (H&E) staining of liver tissues in LPS-induced mice showed distinct histological abnormalities, including liver congestion, hepatocyte swelling, liver rope destruction, and inflammatory cell infiltration (Fig. 1A). According to previous reports [20], the histological scores of the pathological films were obtained, and LPS-induced mice showed higher histological scores compared with mice injected with physiological saline (Fig. 1C). Terminal deoxynucleotidyl transferase dUTP nick-end labeling (TUNEL) staining of livers also showed more apoptotic signals in LPS-induced mice (Fig. 1B). Serum ALT and AST levels were markedly higher after mice were injected with LPS (Fig. 1D, E). F4/80 is a marker of hepatic macrophages and monocyte chemotactic protein-1 (MCP-1) is close to the activation of hepatic macrophages. Myeloperoxidase (MPO) reflects the amount of neutrophils. Immunohistochemical (IHC) staining showed more positive F4/80, MCP-1, and MPO signals in the livers of mice injected with LPS (Fig. 1F, G and Supplementary Fig. S1A), suggesting that more inflammatory cells infiltrated and were activated in the livers of LPS-induced mice, especially hepatic macrophages. Meanwhile, the levels of proinflammatory factors including *TNF-α*, *IL-1β*, and *IL-6* increased in the livers of LPS-induced mice (Fig. 1H), further reflecting the activation of hepatic macrophages.

### ARRB1 is involved in LPS-induced activation of liver macrophages

**Fig. 2:**
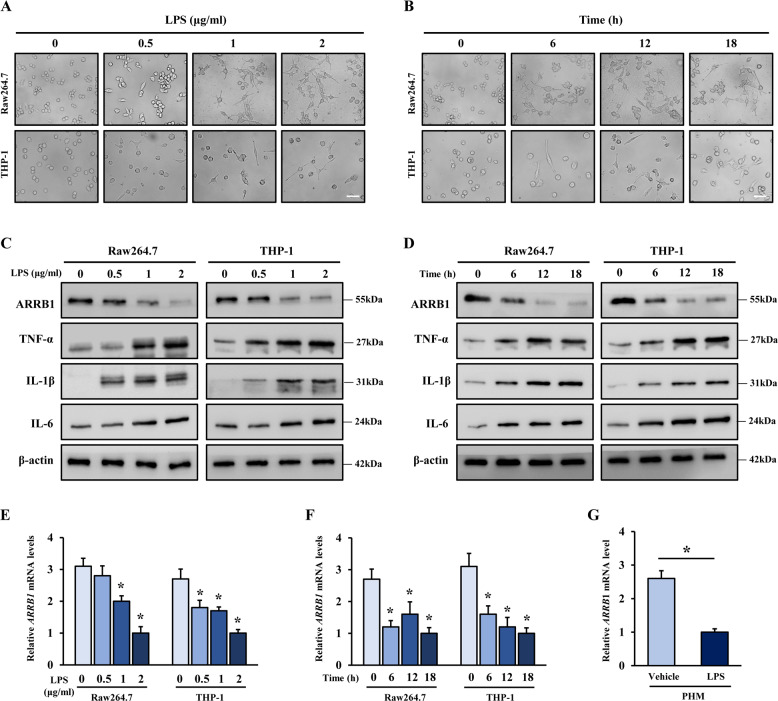
ARRB1 was involved in LPS-induced activation of liver macrophages. **A** Representative morphology images of Raw264.7 and THP-1 cells challenged with LPS at the indicated concentration for 6 h (scale bar: 50 μm). **B** Representative morphology images of Raw264.7 and THP-1 cells treated with LPS (1 μg/ml) were challenged at the indicated times (scale bar: 50 μm). **C** Western blotting analysis of ARRB1, TNF-α, IL-1β, and IL-6 in cells challenged with LPS at the indicated concentrations for 6 h. β-Actin was used as the loading control. **D** Western blotting analysis of ARRB1, TNF-α, IL-1β, and IL-6 in cells with LPS (1 μg/ml) challenge at the indicated times. **E** *ARRB1* mRNA levels in Raw264.7 and THP-1 cells were analyzed at the indicated concentration for 6 h by real-time PCR. *P < 0.05 compared with LPS (0 μg/ml) treatment by one-way ANOVA. **F** *ARRB1* mRNA levels in Raw264.7 and THP-1 cells with LPS (1 μg/ml) challenge at the indicated times were analyzed by real-time PCR. *P < 0.05 compared with vehicle treatment (0 h) by one-way ANOVA. **G** *ARRB1* mRNA levels in mouse primary hepatic macrophages (PHMs) with LPS (1 μg/ml) challenge for 6 h were analyzed by real-time PCR. *P < 0.05 by Student’s *t* test. Data are presented as the mean ± SD of three separate experiments.

Our previous study demonstrated that ARRB1 played a significant role in hepatocyte proliferation and hepatic stellate cell activation [15, 21]. Therefore, we sought to explore whether ARRB1 was involved in LPS-induced activation of liver macrophages in ALI. Two kinds of macrophage cell lines (Raw264.7 and THP-1) were treated with different concentrations of LPS (0, 0.5, 1, and 2 μg/ml) for 6 h. Interestingly, we found that Raw264.7 and THP-1 cells obviously exhibited characteristics of activated macrophages when stimulated with LPS (1 μg/ml), such as increased size, polygonization, and much more and longer pseudopodia (Fig. 2A). Meanwhile, the cells were treated with LPS (1 μg/ml) for different times (0, 6, 12, and 18 h). The results showed that Raw264.7 and THP-1 cells began to exhibit characteristics of activated macrophages when stimulated with LPS for 6 h (Fig. 2B). Moreover, we collected the total proteins from Raw264.7 and THP-1 cells stimulated with LPS at different concentrations and times. The results indicated that the expression level of ARRB1 protein was reduced and the levels of proinflammatory cytokines (TNF-α, IL-1β, and IL-6) increased along with the activation of macrophages (Fig. 2C, D). Consistently, we observed that the transcription of *ARRB1* was reduced following LPS stimulation (Fig. 2E, F). Furthermore, primary hepatic macrophages (PHMs) of WT mice were extracted and treated with LPS (1 μg/ml, 6 h), and the expression level of *ARRB1* messenger RNA (mRNA) was significantly downregulated following LPS stimulation (Fig. 2G). These results implied that ARRB1 may correlate with LPS- induced activation of liver macrophages.

### *ARRB1* deficiency facilitates LPS-induced ALI and activation of liver macrophages

**Fig. 3:**
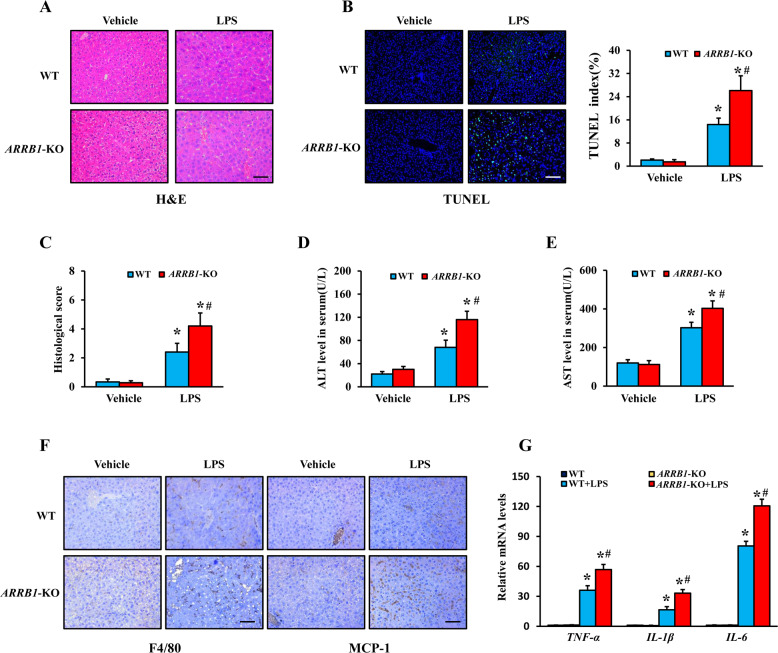
*ARRB1* deficiency aggravated LPS-induced acute liver injury and activation of liver macrophages. **A** Representative H&E staining of livers in vehicle- and LPS-induced *ARRB1* wild-type (WT) and *ARRB1* knockout (KO) mice (scale bar: 50 μm). **B** TUNEL staining of livers in vehicle- and LPS-induced WT and *ARRB1*-KO mice (scale bar: 50 μm), and the positive TUNEL index was scored. **C** Liver histology scoring in vehicle- and LPS-induced WT and *ARRB1*-KO mice. **D**, **E** Serum ALT and AST levels in vehicle- and LPS-induced WT and *ARRB1*-KO mice. **F** F4/80 and MCP-1 staining of livers in vehicle- and LPS-induced WT and *ARRB1*-KO mice (scale bar: 50 μm), and the positive F4/80 and MCP-1 indexes were scored. **G** *TNF-α*, *IL-1β*, and *IL-6* mRNA levels in vehicle- and LPS-induced WT and *ARRB1*-KO mouse liver tissues were analyzed by real-time PCR. All values are the mean ± SD (*n* = 6 in each group). *P < 0.05 compared with the respective vehicle treatment; ^#^*P* < 0.05 compared with LPS-induced WT mice by Student’s *t* test.

Our previous study demonstrated that ARRB1 was involved in chronic liver diseases, such as liver cirrhosis and hepatocellular carcinoma [15, 21]. To confirm the role of ARRB1 in LPS-induced ALI, we generated an LPS (5 mg/kg)-induced ALI model in *ARRB1*-deficient mice (*ARRB1*-KO) and WT mice (Supplementary Fig. S2A, B). H&E-stained liver sections revealed that there were no significant differences in liver tissue structure between WT and *ARRB1*-KO mice without LPS treatment, while following LPS treatment, the livers of *ARRB1*-KO mice exhibited more severe liver congestion, hepatocyte swelling, liver rope destruction, and inflammatory cell infiltration than the livers of WT mice (Fig. 3A). Consistently, LPS-induced *ARRB1*-KO mice showed a higher histological score compared with WT mice injected with LPS (Fig. 3C). TUNEL staining of livers also showed more apoptotic signals in LPS-induced *ARRB1*-KO mice than in LPS-induced WT mice (Fig. 3B). Meanwhile, serum ALT and AST levels were also obviously higher in LPS-induced *ARRB1*-KO mice compared to LPS-induced WT mice (Fig. 3D, E). In addition, we found an increase in F4/80 and MCP-1 signals in the livers of LPS-induced *ARRB1*-KO mice compared to LPS-induced WT mice (Fig. 3F). Furthermore, we tested the expression levels of *TNF-α*, *IL-1β*, and *IL-6* in the liver by real-time PCR, and the results showed higher levels of pro-inflammatory cytokines in *ARRB1*-KO mice with LPS treatment compared to LPS-induced WT mice (Fig. 3G). Together, these results confirmed that *ARRB1* deficiency promoted LPS-induced ALI and activation of liver macrophages.

### Overexpression of *ARRB1* inhibits activation of hepatic macrophages induced by LPS

**Fig. 4:**
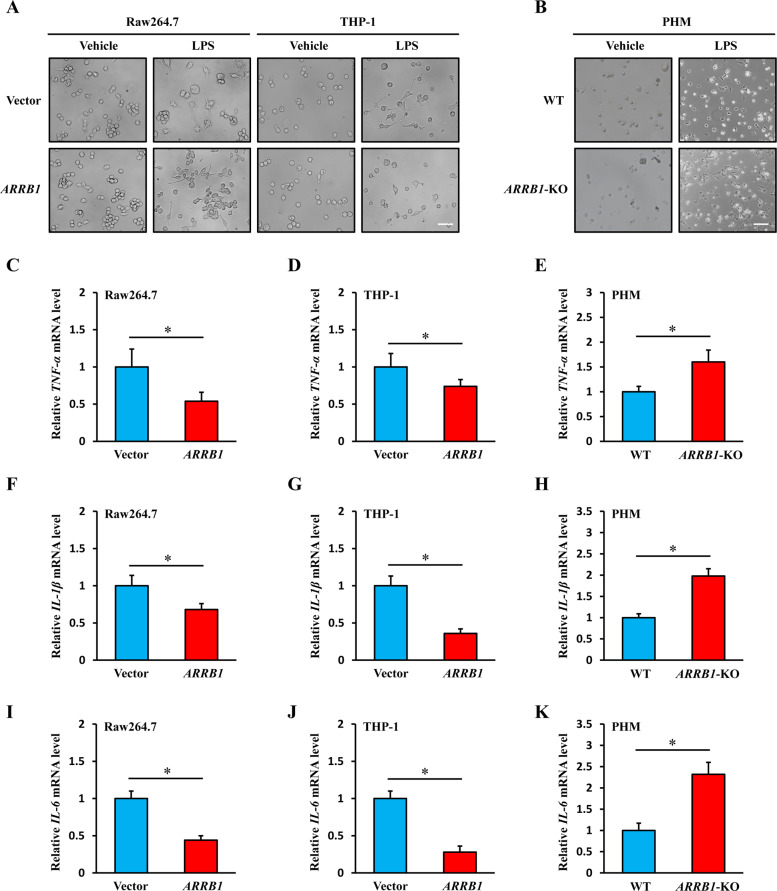
ARRB1 suppressed the activation of hepatic macrophages induced by LPS. **A** Representative morphology images of vector- and *ARRB1*-overexpressing Raw264.7 and THP-1 cells with LPS (1 μg/ml) challenge for 6 h by an inverted microscope (scale bar: 50 μm). **B** Representative morphology images of WT and *ARRB1*-KO mouse primary hepatic macrophages with LPS (0.5 μg/ml) were challenged for 6 h (scale bar: 50 μm). **C**–**K** *TNF-α*, *IL-1β*, and *IL-6* mRNA levels in vector- and *ARRB1*-overexpressing Raw264.7 and THP-1 cells with LPS (1 μg/ml) challenge for 6 h and mouse primary hepatic macrophages with LPS (0.5 μg/ml) challenge for 6 h were analyzed by real-time PCR. Data are presented as the mean ± SD of three separate experiments. *P < 0.05 by Student’s *t* test.

In order to further explore the role of ARRB1 in macrophage activation, we established Raw264.7 and THP-1 cell lines in which *ARRB1* was stably overexpressed by lentivirus (Supplementary Fig. S2C) and examined whether *ARRB1*-overexpressing cells display resistance to macrophage activation. We treated control vector- or *ARRB1*-overexpressing cells with LPS (1 μg/ml) for 6 h and found that vector cells with LPS treatment exhibited evident characteristics of activated macrophages, including increased size, polygonization, and more and longer pseudopodium, but it was reversed in *ARRB1*-overexpressing Raw264.7 and THP-1 cells (Fig. 4A). Then, we stimulated PHMs isolated from WT and *ARRB1*-KO mice with half the concentration of LPS (0.5 μg/ml). Conversely, *ARRB1*-KO mouse PHMs displayed characteristics of activated macrophages accompanied by polygonization and the appearance of pseudopodia, while it cannot be observed in WT mouse PHMs with half the concentration of LPS (Fig. 4B). To further detect cell activation, we tested the levels of *TNF-α*, *IL-1β*, and *IL-6* by real-time PCR in vector- or *ARRB1*-overexpressing Raw264.7 and THP-1 cells treated with LPS (1 μg/ml) treatment for 6 h and mouse PHMs with LPS (0.5 μg/ml) treatment. The results showed that *ARRB1* overexpression inhibited the enhanced expression levels of *TNF-α*, *IL-1β*, and *IL-6* induced by LPS stimulation, but *ARRB1* knockout contributed to the increased levels of these proinflammatory cytokines in mouse PHMs (Fig. 4C–K). These results indicated that overexpression of *ARRB1* suppressed the activation of hepatic macrophages induced by LPS.

### ARRB1 restrains LPS-induced ER stress in hepatic macrophages

**Fig. 5:**
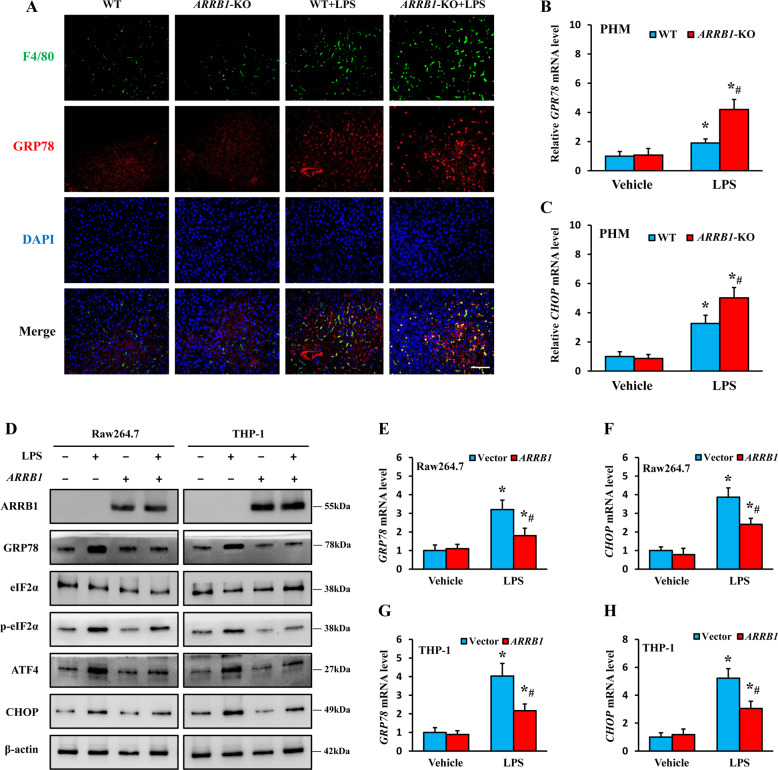
Overexpression of *ARRB1* suppressed LPS-induced ER stress in hepatic macrophages. **A** Double immunofluorescent staining for F4/80 and GRP78 in the indicated mouse liver sections. Nuclei were stained with DAPI in blue. F4/80 was stained green, and GRP78 was visualized red. The merged positive signals of F4/80 and GRP78 are visualized in yellow (scale bar: 50 μm). **B**, **C** *GRP78* and *CHOP* mRNA levels in primary hepatic macrophages with LPS (0.5 μg/ml) challenge for 6 h from WT and *ARRB1*-KO mice were analyzed by real-time PCR. *P < 0.05 compared with the respective vehicle treatment; ^#^*P* < 0.05 compared with LPS-induced WT mice by Student’s *t* test. **D** Protein expression levels of ARRB1, GRP78, eIF2α, p-eIF2α, CHOP, and ATF4 in *ARRB1*-overexpressing Raw264.7 and THP-1 cells with or without LPS (1 μg/ml) challenge for 6 h. **E**–**H** *GRP78* and *CHOP* mRNA levels of *ARRB1*-overexpressing Raw264.7 and THP-1 cells with LPS (1 μg/ml) were challenged for 6 h and analyzed by real-time PCR. *P < 0.05 compared with the respective vehicle treatment; ^#^*P* < 0.05 compared with vector cells following LPS treatment by Student’s *t* test. Data are presented as the mean ± SD of three separate experiments.

ER stress signaling has been reported to be a vital promotor of LPS-induced ALI [22]. We next determined whether ER stress was related to ARRB1 in LPS-induced ALI. Indeed, the evidence obtained by double immunofluorescent staining for F4/80 and GRP78, a core protein in ER stress, revealed that most of the elevated positive signals overlapped in the liver sections of LPS-induced *ARRB1*-KO mice instead of LPS-induced WT mice (Fig. 5A), suggesting that ER stress in LPS-induced ALI may be regulated by ARRB1. Then, we tested the mRNA levels of *GRP78* and *CHOP* in mouse PHMs stimulated with LPS (0.5 μg/ml) stimulation for 6 h, and the results showed higher mRNA levels of *GRP78* and *CHOP* in *ARRB1*-KO mouse PHMs treated with LPS than in PHMs from WT mice (Fig. 5B, C). To thoroughly define the relationship between ER stress and ARRB1, vector- or *ARRB1*-overexpressing Raw264.7 and THP-1 cells were stimulated with or without LPS (1 μg/ml). We observed apparently increased levels of GRP78, p-eIF2α, CHOP, and ATF4 in the LPS-treated vector cells (Fig. 5D), which implied that ER stress had occurred. However, the trend of the increase was weakened in the LPS-treated *ARRB1*-overexpressing cells (Fig. 5D), suggesting that ARRB1 may be a negative regulator of LPS-induced ER stress. Consistently, the mRNA levels of *GRP78* and *CHOP* were enhanced in the Raw264.7 and THP-1 cells challenged with LPS, but this effect was reversed in *ARRB1*-overexpressing Raw264.7 and THP-1 cells with LPS stimulation (Fig. 5E–H). Overall, ARRB1 notably inhibited LPS-induced ER stress in hepatic macrophages.

### Inhibition of ER stress disturbs ARRB1-modulated activation of liver macrophages in vivo and in vitro

**Fig. 6:**
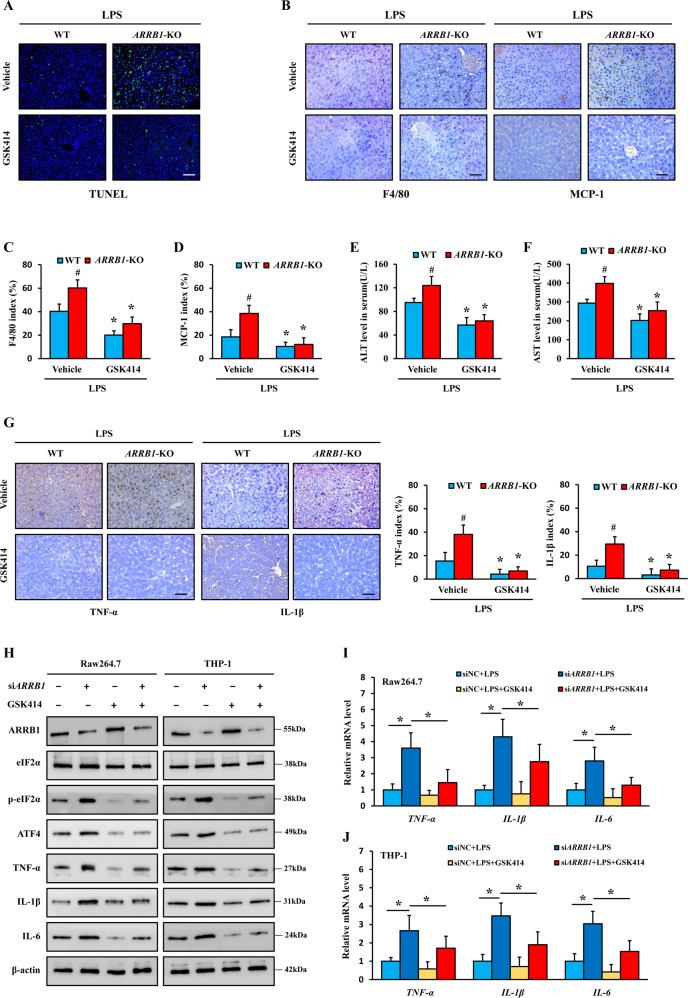
Blockage of ER stress abrogated ARRB1-modulated activation of liver macrophages in vivo and in vitro. Mice were intraperitoneally administered LPS injection (5 mg/kg) 8 h after gavage with GSK414 (150 mg/kg) or vehicle solution. *n* = 6 in each group. **A** TUNEL staining of livers in LPS-induced mice with or without GSK414 challenge (scale bar: 100 μm). **B**–**D** F4/80 and MCP-1 staining of livers in LPS-induced mice with or without GSK414 challenge (scale bar: 50 μm), and the positive F4/80 and MCP-1 indexes were scored. **E**, **F** Serum ALT and AST levels in LPS-induced mice with or without GSK414 challenge. **G** TNF-α and IL-1β staining of livers in LPS-induced mice with or without GSK414 challenge (scale bar: 50 μm), and the positive TNF-α and IL-1β indexes were scored. **C**–**G** *P < 0.05 compared with the respective vehicle treatment; ^#^*P* < 0.05 compared with LPS-induced WT mice by Student’s *t* test. **H**–**J** Raw264.7 and THP-1 cells were challenged with LPS (1 μg/ml) for 6 h with or without GSK414 (1 μM) treatment after knockdown of *ARRB1* by siRNA. **H** Western blotting analysis of the indicated protein expression. **I**, **J** *TNF-α*, *IL-1β*, and *IL-6* mRNA levels in Raw264.7 and THP-1 cells in the indicated treatments were analyzed by real-time PCR. *P < 0.05 by Student’s *t* test. Data are presented as the mean ± SD of three separate experiments.

The preceding data show that ARRB1 suppressed ER stress in LPS-induced ALI and that downregulation of ARRB1 exacerbated LPS-induced activation of liver macrophages. In order to further determine whether ARRB1 regulates LPS-induced activation of liver macrophages through ER stress signaling, WT or *ARRB1*-KO mice were injected intraperitoneally with LPS (5 mg/kg) 8 h after being administered with GSK2606414 (GSK414) (150 mg/kg) or vehicle solution by oral gavage. GSK414 is a canonical inhibitor of ER stress that selectively inhibits PERK. Treatment with GSK414 in vivo and in vitro effectively impeded the phosphorylation of PERK [23] and suppressed the activation of the eIF2α/ATF4/CHOP signaling pathway (Fig. 6H). As expected, the application of GSK 414 obviously ameliorated the disease activity of ALI induced by LPS, even in LPS-induced *ARRB1*-KO mice (Fig. 6A). Additionally, inhibition of ER stress by GSK414 attenuated F4/80 and MCP-1 accumulation in the livers of LPS-induced WT and *ARRB1*-KO mice (Fig. 6B–D), implying that ER stress is involved in ARRB1-mediated activation of liver macrophages. Serum ALT and AST levels were reduced in LPS-induced *ARRB1*-KO mice following GSK414 treatment (Fig. 6E, F), suggesting that suppression of ER stress may alleviate a more severe liver inflammatory response due to *ARRB1* deficiency. Furthermore, IHC detection revealed that the inhibition of ER stress in LPS-induced *ARRB1*-KO mice led to the downregulation of TNF-α and IL-1β (Fig. 6G). On the other hand, we found that knockdown of *ARRB1* by small interfering RNA (siRNA) enhanced the mRNA and protein expression levels of TNF-α, IL-1β, and IL-6 in Raw264.7 and THP-1 cells with LPS treatment, but suppression of ER stress abolished the effect of ARRB1 on LPS-induced activation of liver macrophages (Fig. 6H–J). Together, these results confirmed that ARRB1 regulates the activation of liver macrophages induced by LPS through inhibition of ER stress.

## Discussion

**Fig. 7:**
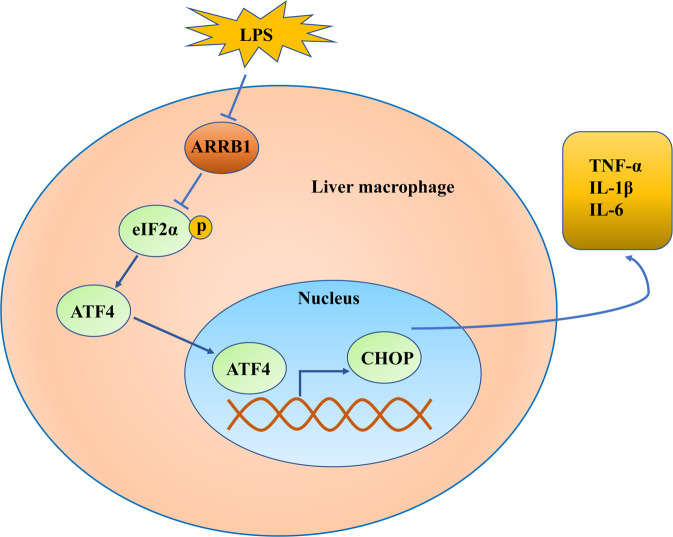
Summary of the proposed regulatory mechanism of ARRB1 in this study. ARRB1 suppresses LPS-induced release of inflammatory cytokines in response to activated macrophages. ARRB1 regulates the ER stress signaling pathway by inhibiting the phosphorylation of eIF2α and downstream ATF4 and CHOP expression in liver macrophages.

ALI is a disease caused by multiple etiologies with high morbidity and mortality and is characterized by persistent and excessive inflammatory cascades and serious inflammatory damage to hepatocytes. Among these etiologies such as virus infection and side effects of drug and alcohol abuse, sepsis is a particularly common and severe etiology that leads to multiple organ dysfunction syndromes and poor prognosis [24]. Nevertheless, to date, the mechanism of ALI pathogenesis has not been completely understood. Hence, exploring targeting molecules that involve and determine the occurrence and development of the disease may be helpful for ALI therapy. In this study, we demonstrated that ARRB1 protected against LPS-induced ALI through regulation of macrophage activation via ER stress (Fig. 7).

LPS, a component of gram-negative bacteria, stimulates innate inflammatory and immune responses via pattern recognition receptors, particularly Toll-like receptors (TLRs), and is involved in the pathogenesis of liver injury in patients with severe bacterial infection [6]. Here, we successfully established a mouse model of ALI induced by LPS for the study of sepsis-associated liver injury and observed massive inflammatory cell infiltration in hepatic sinuses with congestion following LPS treatment, along with increased hepatocyte apoptosis and serum ALT and AST. Interestingly, we found that the expression level of ARRB1 was reduced in LPS-induced macrophages. Zeng et al. showed that upregulation of ARRB1 mediated TLR2 activation-induced apoptosis resulting in increased histone H4 acetylation in bone marrow CD34^+^ cells [25]. Moreover, TLR4 and CD44 activation following IL-1β stimulation contributed to downstream ARRB1/NF-κB activation and inflammatory mediator transcription [26]. These evidences suggest that TLRs may provide research clues to explain the effects of LPS on ARRB1 in different cells.

Actually, existing studies have reported that ARRB1 not only responds to the termination of G-protein activation as a classic adaptor protein but also participates in the mediation of the inflammatory response through multiple signal transduction pathways. Preceding research from our team revealed that ARRB1 alleviated acute pancreatitis via repression of NF-κBp65 activation [27]. A recent study showed that ARRB1 inhibited nonalcoholic steatohepatitis progression by promoting growth differentiation factor 15 maturation [28]. These studies indicate that ARRB1 directly or indirectly regulates inflammatory responses in various disease models. In this study, we found that the absence of ARRB1 exacerbated the activation of hepatic macrophages, accounting for disordered inflammatory cascades and more severe AKI. On the other hand, in previous studies, we found that the expression level of ARRB1 was enhanced in the livers of mice intraperitoneally injected with CCl4 or diethylnitrosamine (DEN) to induce AKI and that TNF-α stimulation facilitated ARRB1 expression in hepatocytes [15]. Here, we discovered that ARRB1 modulated the release of proinflammatory cytokines including TNF-α, IL-1β, and IL-6 in hepatic macrophages, which means that ARRB1-mediated release of inflammatory cytokines, in turn, may regulate ARRB1 expression. Consistently, ARRB1 is a protective factor in CCl_4_ or DEN-induced AKI as well as LPS-induced AKI. Hence, combined with previous studies, we hypothesized that ARRB1 has different functions in hepatocytes and macrophages in LPS-induced AKI. In fact, ARRB1 is also removed from hepatocytes in *ARRB1*-KO mice, and a lack of ARRB1 blocks the proliferation of liver cells in inflammation-mediated hepatocellular carcinogenesis [15]. In this research, we also found that the lack of ARRB1 aggravated hepatocyte apoptosis, which may not be due only to the damage we found caused by inflammatory cytokines. Given this information, the role of ARRB1 in hepatocytes and its influence on ALK deserve further investigation.

Activation of macrophages is mediated by signaling cascades downstream of TLR and cytokine receptors, such as PI3K/Akt, NF-κB, and mitogen-activated protein kinase signaling [29]. Most G-protein-mediated signal-transduction pathways are deemed to be relevant to the stimulation of macrophages by activators or immunomodulators [30]. ER stress is well described as a crucial mediator of inflammatory injury and triggers macrophage activation and even hepatocyte apoptosis if the inflammatory injury continuously exists. To verify whether this triggering of macrophage activation and even hepatocyte apoptosis also occurred in LPS-induced ALI, we administered GSK414, a specific inhibitor of ER stress, in vivo and in vitro, and consistent with previous studies, our results showed that the application of GSK414 effectively suppressed ER stress and subsequent macrophage activation by preventing the activation of the eIF2α/ATF4/CHOP signaling pathway. Moreover, we confirmed that ER stress was an indispensable modulator of ARRB1-mediated suppression of macrophage activation. However, blocking ER stress did not affect the expression level of ARRB1, though interference with ARRB1 attenuated ER stress. Additionally, a previous study found that ARRB1 restrained upregulated ER stress/p53 via repressing p-p65/inducible nitric oxide synthase in portal hypertensive gastropathy [16]. Indeed, genetic abnormalities and the aberrant inflammatory microenvironment of the liver respond to the induction of ER stress in macrophages of ALI. Abundant genetic studies have revealed primary aberrances in several ER homeostasis-associated genes, such as carboxylesterase 2, sirtuin 1, and interferon regulatory factor 3, in AKI patients [31–33]. Combined with preceding studies from our team, ARRB1 may be a potential ER homeostasis-associated gene.

In conclusion, our findings revealed that *ARRB1* deficiency aggravated LPS-induced ALI resulting from excessive activation of macrophages. Blocking ER stress abolished more severe macrophage activation and exacerbated ALI due to ARRB1 downregulation. In brief, ARRB1 suppresses the activation of hepatic macrophages via regulating ER stress in LPS-induced ALI, and ARRB1, especially ARRB1-mediated ER stress may be a promising therapeutic target for ALI.

## Materials and methods

### Treatment of mice

All animal experiments in this study were conducted with the approval of the Institutional Animal Care and Use Committee at Third Affiliated Hospital of Sun Yat-Sen University. *ARRB1*-KO mice were gifts from Dr. Robert J Lefkowitz, Duke University Medical Center, Durham, NC, USA. All *ARRB1*-WT and *ARRB1*-KO mice bred on a C57BL/6 background were generated by heterozygote intercrosses. The mice were housed in microisolator cages under controlled conditions at room temperature with 50% humidity and a 12-/12-h light–dark cycle (lights on at 8:00 a.m.) with food and sterilized water ad libitum. To establish the ALI model, overnight fasting 6-week-old male mice were intraperitoneally injected with LPS (Sigma, St. Louis, MO) at 5 mg/kg. In the control trial, mice were intraperitoneally injected with the same volume of physiological saline. To inhibit ER stress in vivo, 6-week-old male mice were administered GSK414 (Selleck, Shanghai, China) suspended in vehicle solution containing 0.5% hydroxypropyl methylcellulose and 0.1% Tween-80 in water at pH 4.8 (150 mg/kg body weight) by oral gavage 8 h before intraperitoneal injection of LPS (5 mg/kg). The control mice were injected only with an equal dose of LPS. The mice were sacrificed 6 h later through carbon dioxide inhalation used as the method of euthanasia.

### Cell culture and treatment

Cell lines were purchased from the American Type Culture Collection. Raw264.7 cells were cultured in Dulbecco’s modified Eagle’s medium (DMEM) (Gibco BRL, Rockville, MD, USA) containing 10% heat-inactivated fetal bovine serum (FBS). THP-1 cells were cultured in RPMI-1640 medium (Gibco BRL, Rockville, MD, USA) containing 10% FBS and 0.05 mM β-mercaptoethanol (Sigma, St. Louis, MO). To induce THP-1 cell differentiation into macrophages, phorbol 12-myristate 13-acetate (Selleck, Shanghai, China, 10 ng/ml) was added to THP-1 cells for 48 h before other treatments. All cells were cultured in an incubator with 5% CO_2_ at 37 °C. For drug intervention, Raw264.7 cells and THP-1 cells were treated with the indicated concentration of LPS (Sigma, St. Louis, MO), with or without 1 μM GSK414 (Selleck, Shanghai, China) for the indicated times.

### Establishment of cell lines and interference with *ARRB1*

*ARRB1*-overexpressing lentiviruses were purchased from GeneChem (Shanghai, China). To establish stable *ARRB1*-overexpressing Raw264.7 cells and THP-1 cell lines, we infected the cells with the *ARRB1* lentivirus according to the instructions and Raw264.7 cells and THP-1 cells were selected by puromycin (2 μg/ml). To silence the *ARRB1* gene, *ARRB1* siRNA was transfected into cells according to the instructions of Lipofectamine 3000 (Thermo Fisher Scientific, USA). The *ARRB1* siRNA sequence was 5′-GTCACCAACAACACCAACA-3′ (Gene Pharma, China).

### RNA extraction and real-time PCR for gene expression

For quantitative real-time PCR, total RNA was extracted from freshly frozen liver tissues or cells by TRIzol (Promega, Madison, WI, USA), and then first-strand complementary DNA synthesis was performed by a Reverse Transcription Kit (TOYOBO, Japan) according to the manufacturer’s instructions. The mRNA levels of relevant genes were detected by a Mini Opticon Real-time PCR System (Bio-Rad, Hercules, CA, USA) with SYBR Green (Invitrogen, USA). β-Actin was used as a normalized control in the expression level analysis of relevant genes. Primer sequences of relevant genes are provided in Table 1.

**Table 1:** Primer sequences used in real-time PCR analysis.

Human	Primer sequences
*ARRB1*	5′-GCGAGCACGCTTACCCTTT-3′ (sense) 5′-CAAGCCTTCCCCGTGTCTTC-3′ (antisense)
*TNF-α*	5′-GTTCCTCAGCCTCTTCTCCT-3′ (sense) 5′-ACAACATGGGCTACAGGCTT-3′ (antisense)
*IL-1β*	5′-TCCCCAGCCCTTTTGTTGA-3′ (sense) 5′-TTAGAACCAAATGTGGCCGTG-3′ (antisense)
*IL-6*	5′-ATGTCTGAGGCTCATTCTGC-3′ (sense) 5′-GCGGCTACATCTTTGGAATC-3′ (antisense)
*GRP78*	5′-GAGACAGTGGGTAGGGAAGTGC-3′ (sense) 5′-GAACAAATCGGAACAATGCTAA-3′ (antisense)
*CHOP*	5′-CCACTCTTGACCCTGCTTC-3′ (sense) 5′-CCACTCTGTTTCCGTTTCC-3′ (antisense)
*β-actin*	5′-GTCTTCCCCTCCATCGTG-3′ (sense) 5′-AGGGTGAGGATGCCTCTCTT-3′ (antisense)
Mouse	Primer sequences
*ARRB1*	5′-CCGAGGACAAGAAGCCACTGA-3′ (sense) 5′-AGAGTGACTGAGCATGGAAGGT-3′ (antisense)
*TNF-α*	5′-CTTCATCACCTATCCCTCGAC-3′ (sense) 5′-CTGGCTATTTGCTTCTTGTCCT-3′ (antisense)
*IL-1β*	5′-CTTTGAAGTTGACGGACCC-3′ (sense) 5′-TGAGTGATACTGCCTGCCTG-3′ (antisense)
*IL-6*	5′-TTGTGCAATGGCAATTCTGA-3′ (sense) 5′-CGGACTCTGGCTTTGTCTTTCT-3′ (antisense)
*GRP78*	5′-GCCGAGGAGGAGGACAAGAA-3′ (sense) 5′-ACACACCGACGCAGGAATAG-3′ (antisense)
*CHOP*	5′-CCCTCGCTCTCCAGATTCC-3′ (sense) 5′-TCTCCTTCATGCGTTGCTT-3′ (antisense)
*β-actin*	5′-GGCTGTATTCCCCTCCATCG-3′ (sense) 5′-CCAGTTGGTAACAATGCCATGT-3′ (antisense)

### Protein extraction and Western blot

Total proteins were extracted from fresh cell lysates by RIPA buffer and examined by Western blot. Antibodies against ARRB1 (Cell Signaling Technology, 12697), GRP78 (Cell Signaling Technology, 3177), eIF2α (Cell Signaling Technology, 5324), p-eIF2α (Cell Signaling Technology, 3398), CHOP (Cell Signaling Technology, 2895), TNF-α (Abclonal, A11534), IL-1β (Abclonal, A19635), IL-6 (Abclonal, A0286), and β-actin (Sigma-Aldrich, A5441) were used as primary antibodies. ECL chemiluminescence (Thermo Fisher Scientific, Waltham, MA, USA) was used for signal visualization analysis. β-Actin was used as a normalized control in the expression level analysis of each target protein.

### Analysis of apoptosis

TUNEL staining was performed using the TUNEL Apoptosis Assay Kit (Beyotime, Shanghai, China) according to the manufacturer’s instructions. The apoptotic index was calculated by dividing the number of apoptotic cells by the total number of cells in the livers of at least 20 randomly selected fields (×200). Six mice from each group were studied.

### IHC and immunofluorescent staining

For histological analysis, formalin-fixed liver tissues were embedded in paraffin, and 3 mm histological sections obtained from paraffin-embedded liver tissues were subjected to H&E and IHC staining. Liver sections were incubated with 3% hydrogen peroxide to eliminate endogenous peroxidase activity. Antigen was retrieved in boiling EDTA buffer (pH 8.0) under high temperature and pressure for 3 min. For IHC staining, primary antibodies against F4/80 (Cell Signaling Technology, 70076), MPO (Abcam, ab208670), TNF-α (Abclonal, A11534), IL-1β (Abclonal, A19635), and MCP-1 (Abclonal, A7277) were used. For double immunofluorescence staining, sections were simultaneously incubated with primary antibodies against F4/80 (rabbit monoclonal, Cell Signaling Technology, 70076) and GRP78 (mouse monoclonal, Proteintech, 66574). Specific Alexa 488 (A11008) and Alexa 594 (A11032) fluorescence-conjugated secondary antibodies (Invitrogen) were used in the dark, and 4′,6-diamidino-2-phenylindole (Invitrogen, D1306) was used to stain the cell nuclei. Images were analyzed using a fluorescence microscope.

### Histopathology scoring of liver injury

Liver tissues were fixed and embedded, and the sections were subjected to H&E staining for subsequent histological assessment. Histological scores were evaluated blindly based on the degree of hemorrhage as previously described [20] in ten randomly magnified fields (×200). Six mice from each group were studied.

### Serum ALT and AST measurements

Serum ALT and AST levels were determined in mouse serum using ALT and AST Detection Kits (Kehua Biology, Shanghai, China) by an automatic biochemical analyzer.

### Isolation of PHMs

The protocol of isolation of murine PHMs was performed according to previous reports [20]. The livers of mice were perfused in situ with collagenase type IV (Sigma-Aldrich, St. Louis, MO, USA) until liver cells could easily be stripped from the liver capsule. The digested liver tissues were passed through a 100-mm mesh filter to gain a cell suspension. Next, the cell suspension was centrifuged at 50 × *g*/min for 1 min, and the supernatant was collected for subsequent isolation. Nonparenchymal cells were isolated at the interface in a discontinuous gradient of 25 and 50% Percoll (Solarbio, Beijing, China) in phosphate-buffered saline salt solution and plated in 24-well plates. Nonadherent cells were removed by replacing the fresh culture medium 1 h later to eliminate other nonparenchymal cells and purify PHMs. Adherent macrophages were continuously cultured with DMEM containing 10% FBS, 100 U/ml penicillin, and 0.1 mg/ml streptomycin (Gibco BRL, Rockville, MD, USA). The cells followed by the indicated drug treatment were photographed by an inverted microscope.

### Statistical analyses

All results were expressed as the mean ± SD. The data were analyzed using SPSS 22.0 and Microsoft Excel. Normal distribution was assumed with regard to small sample sizes. To compare the differences of groups, a two-tailed Student’s *t* test or one-way analysis of variance was applied. Differences were considered to be statistically significant when *P* < 0.05. All trials were individually repeated at least twice with consistent results.

## Supplementary information

supplementary figure 1

supplementary figure 2

## Data Availability

The datasets used and/or analyzed during the current study are available from the corresponding author on reasonable request.
