# Supplementary figures and images for: ARRB1 suppresses the activation of hepatic macrophages via modulating endoplasmic reticulum stress in lipopolysaccharide-induced acute liver injury
Source: Cell Death Discov. 2021 Aug 28;7:223. doi: 10.1038/s41420-021-00615-9 (PMC8403172; doi:10.1038/s41420-021-00615-9)

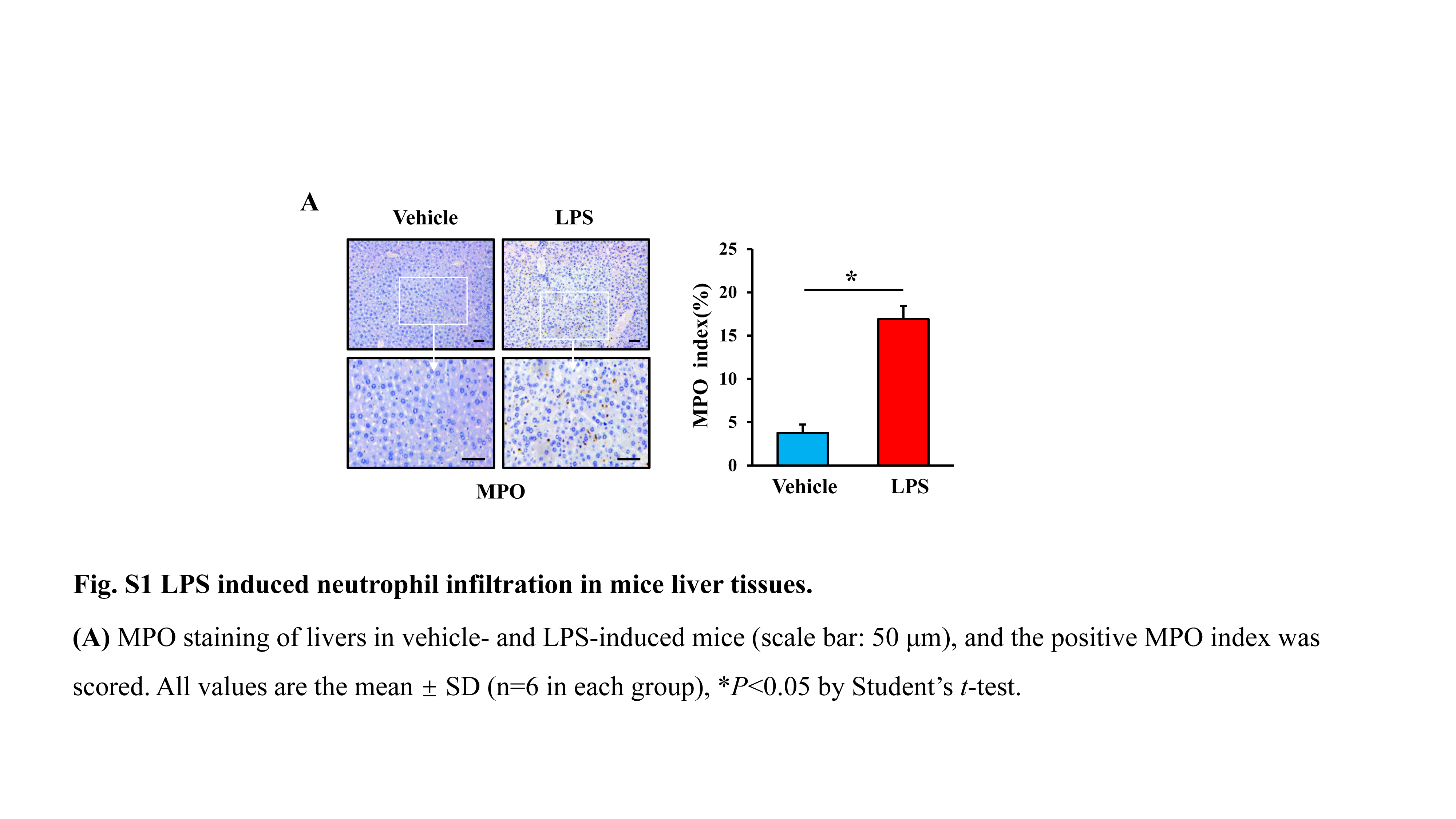

Supplement: Supplementary file 1 — supplementary figure 1 [file 41420_2021_615_MOESM1_ESM.tif]

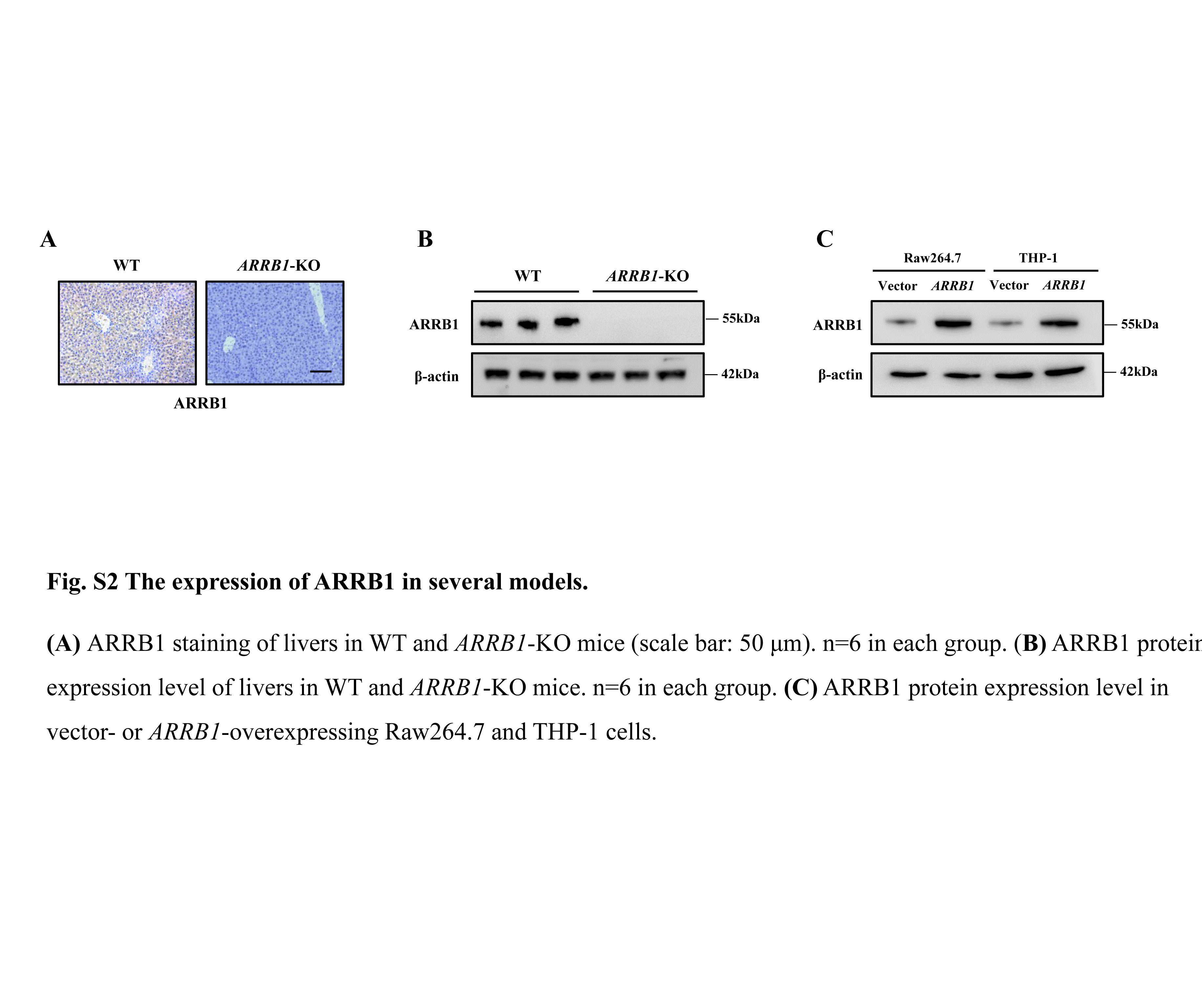

Supplement: Supplementary file 2 — supplementary figure 2 [file 41420_2021_615_MOESM2_ESM.tif]
